# Supplementary material for: Genomic signatures of different adaptations to environmental stimuli between wild and cultivated Vitis vinifera L
Source: Hortic Res. 2018 Jul 1;5:34. doi: 10.1038/s41438-018-0041-2 (PMC6026492; doi:10.1038/s41438-018-0041-2)
Supplement: Supplementary file 1 — GENOMIC SIGNATURES OF DIFFERENT ADAPTATIONS TO ENVIRONMENTAL STIMULI BETWEEN WILD AND CULTIVATED Vitis vinifera L [file 41438_2018_41_MOESM1_ESM.docx]

## Supplementary Information Figs S1-S13; Tables S1, S2, S4-S6 and Note S1.

**GENOMIC SIGNATURES OF DIFFERENT ADAPTATIONS TO ENVIRONMENTAL STIMULI BETWEEN WILD AND CULTIVATED *Vitis vinifera* L.**

*Annarita Marrano, Diego Micheletti, Silvia Lorenzi, David Neale, M. Stella Grando*

The following Supplementary Information is available for this article:

**Figure S1** Distribution of minor allele frequency (MAF) within *sativa* and *sylvestris* subgroups for the SNP datasets obtained with the Vitis20K chip (a) and the RAD-seq (b) assays.


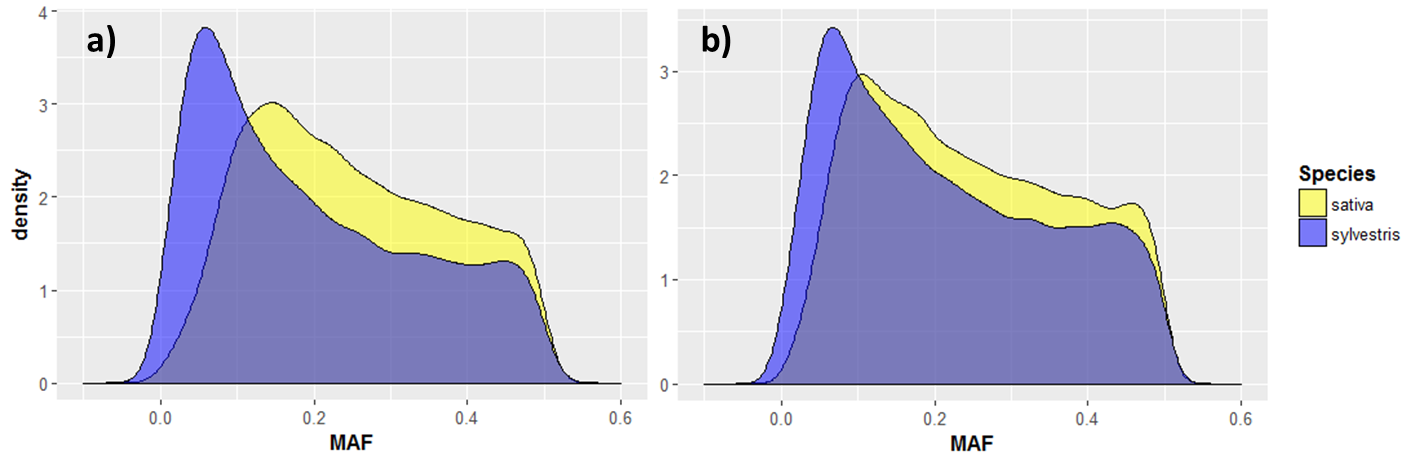


**Figure S2** LD plot (GOLD heatmap) base on r2 values obtained with Haploview v4.1 for each chromosome within the *sativa* subgroup (red = high r2; blue = low r2).
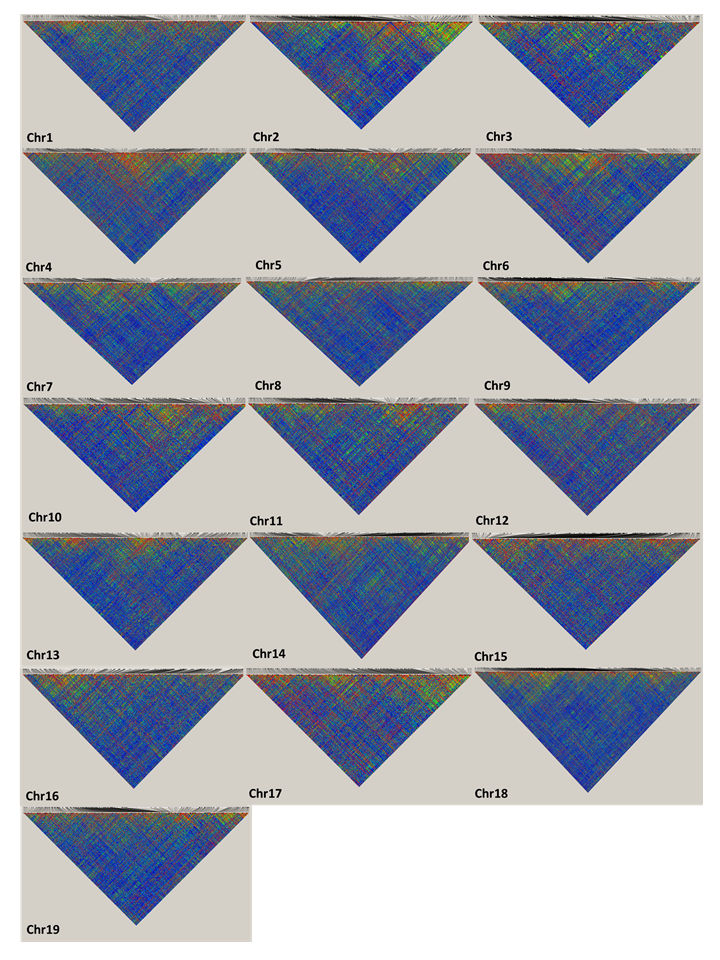


**Figure S3** LD plot (GOLD heatmap) base on r2 values obtained with Haploview v4.1 for each chromosome within the *sylvestris* subgroup (red = high r2; blue = low r2).
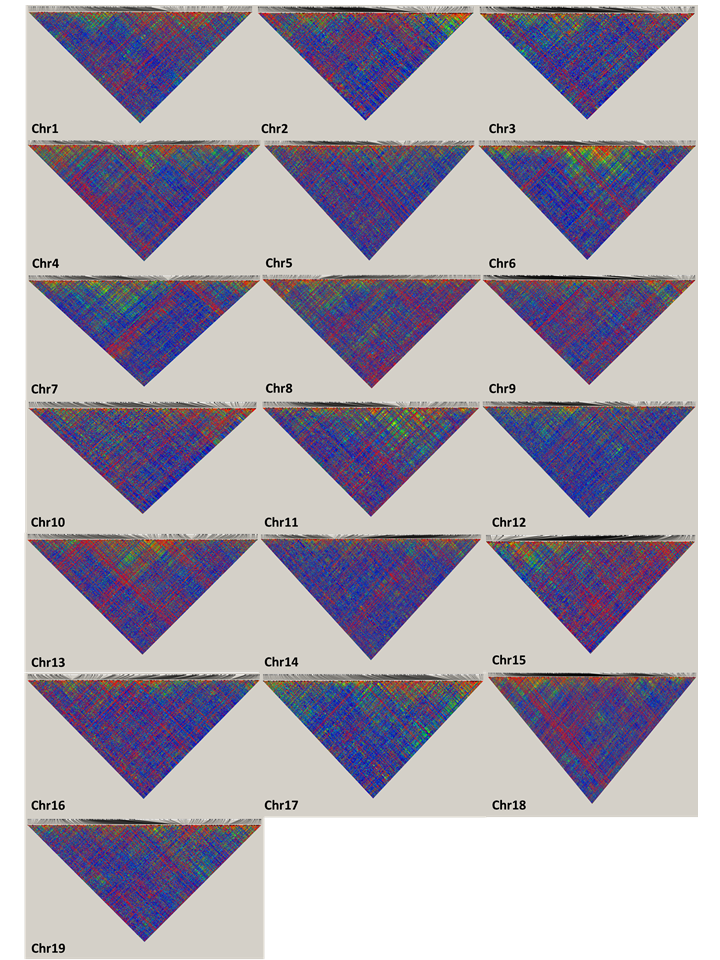


**Figure S4** empirical distribution of FST values across the whole genome between wild and cultivated grapevines. The area shaded in red indicated the top 5% of FST values. The average and standard deviation (sd) of the 95th percentiles of FST values gained over 1,000 permutations are also reported.


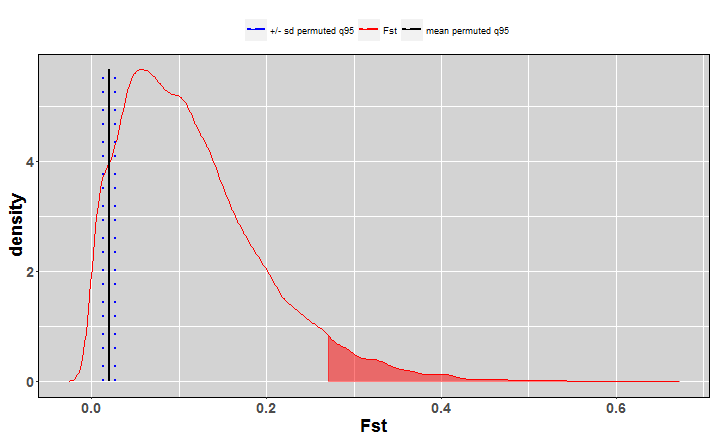


**Figure S5** **:** correlation analysis between phenotypic data collected in 2012 and 2013 for each trait.


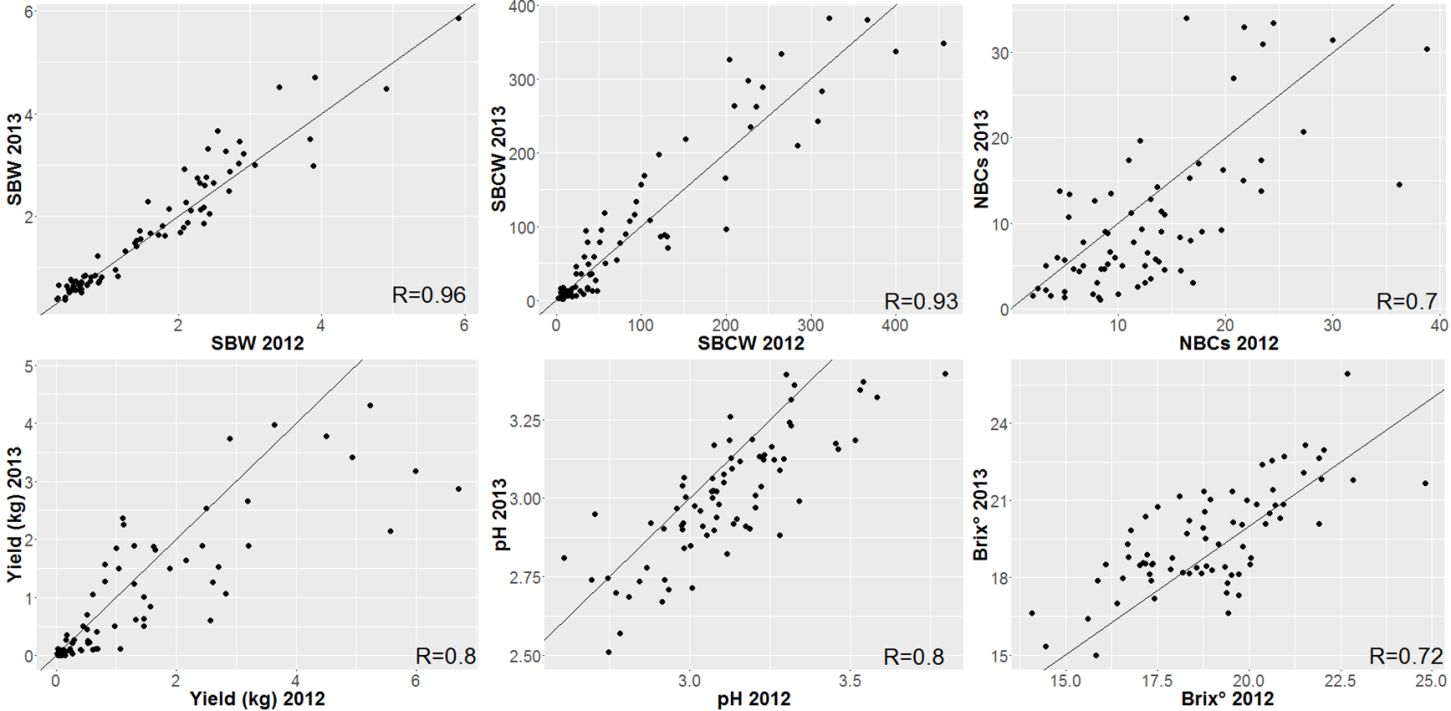


**Figure S6** **:** distribution of the average values per each trait in cultivated and wild accessions separately.


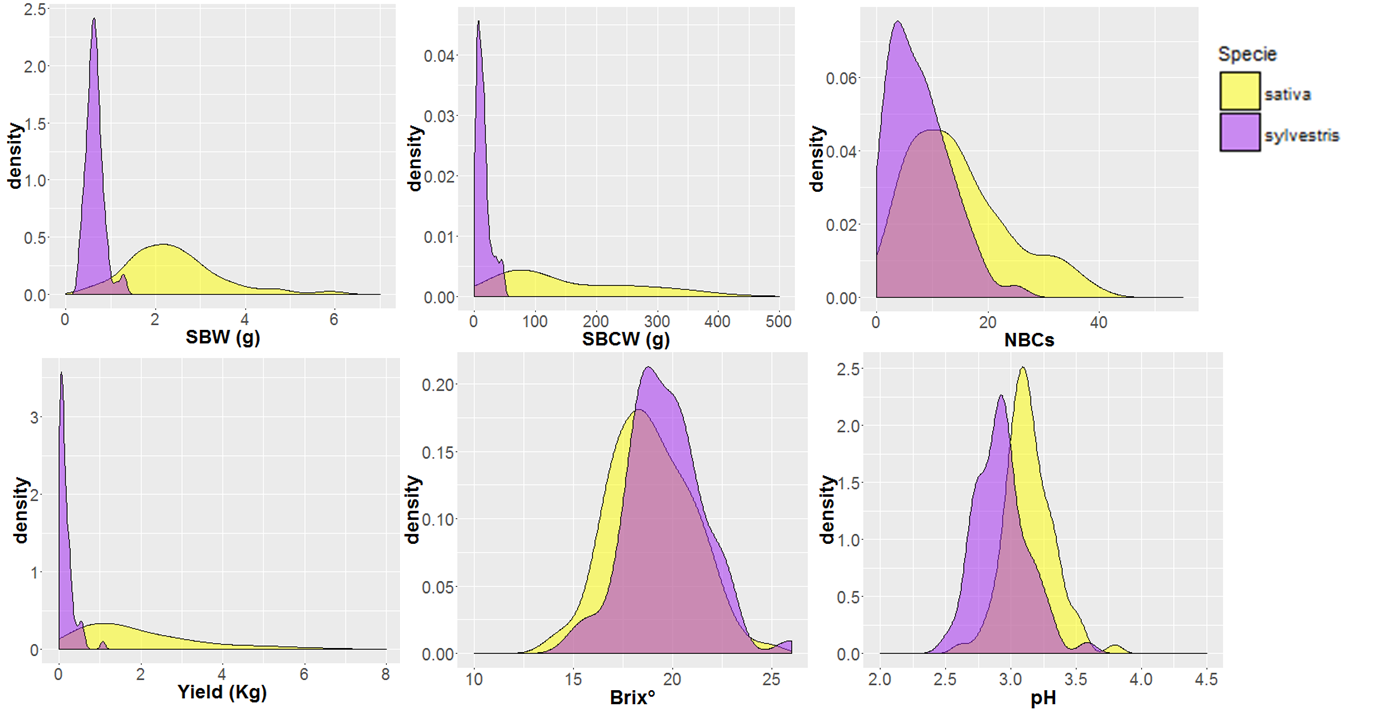


**Figure S7** **:** distribution of the average values per each trait in the two measurements year separately.


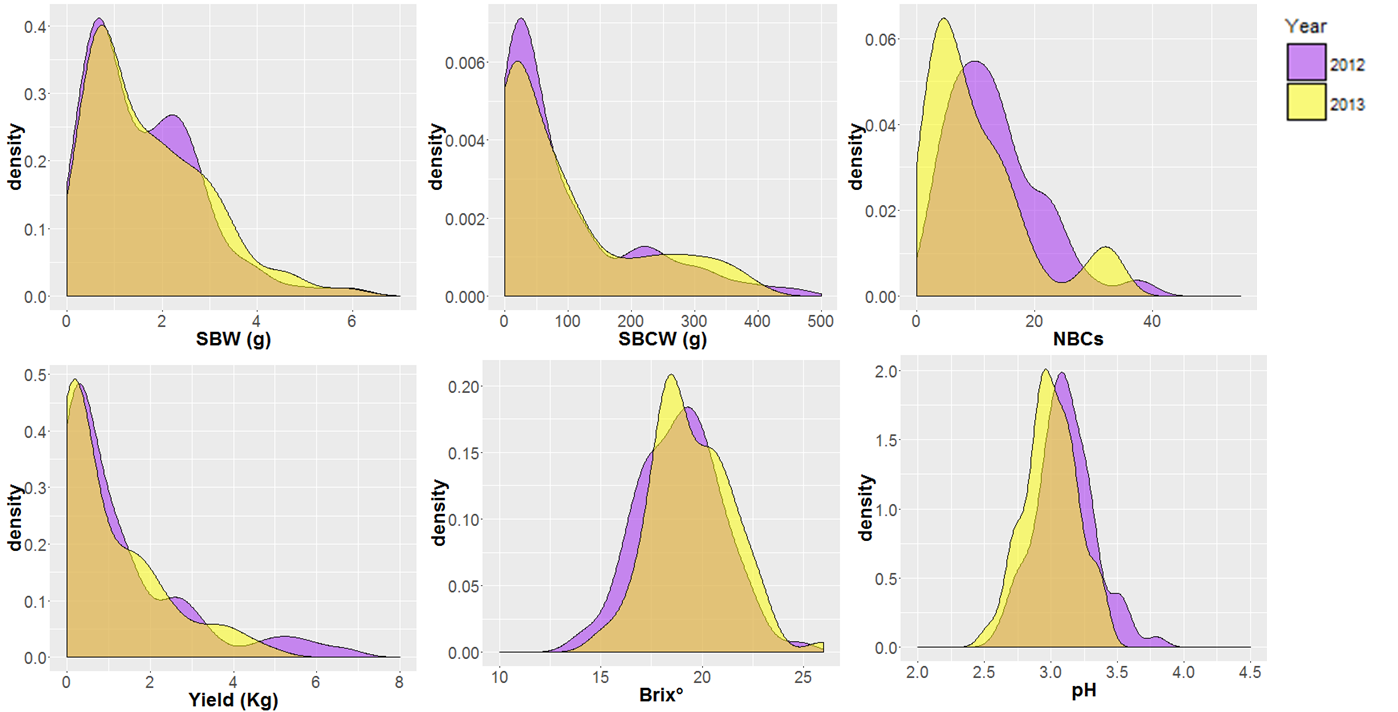


**Figure S8** **:** Q-Q plot of GLM, MLM (Q+K) and MLM (K) models used for GWAS test for all traits.


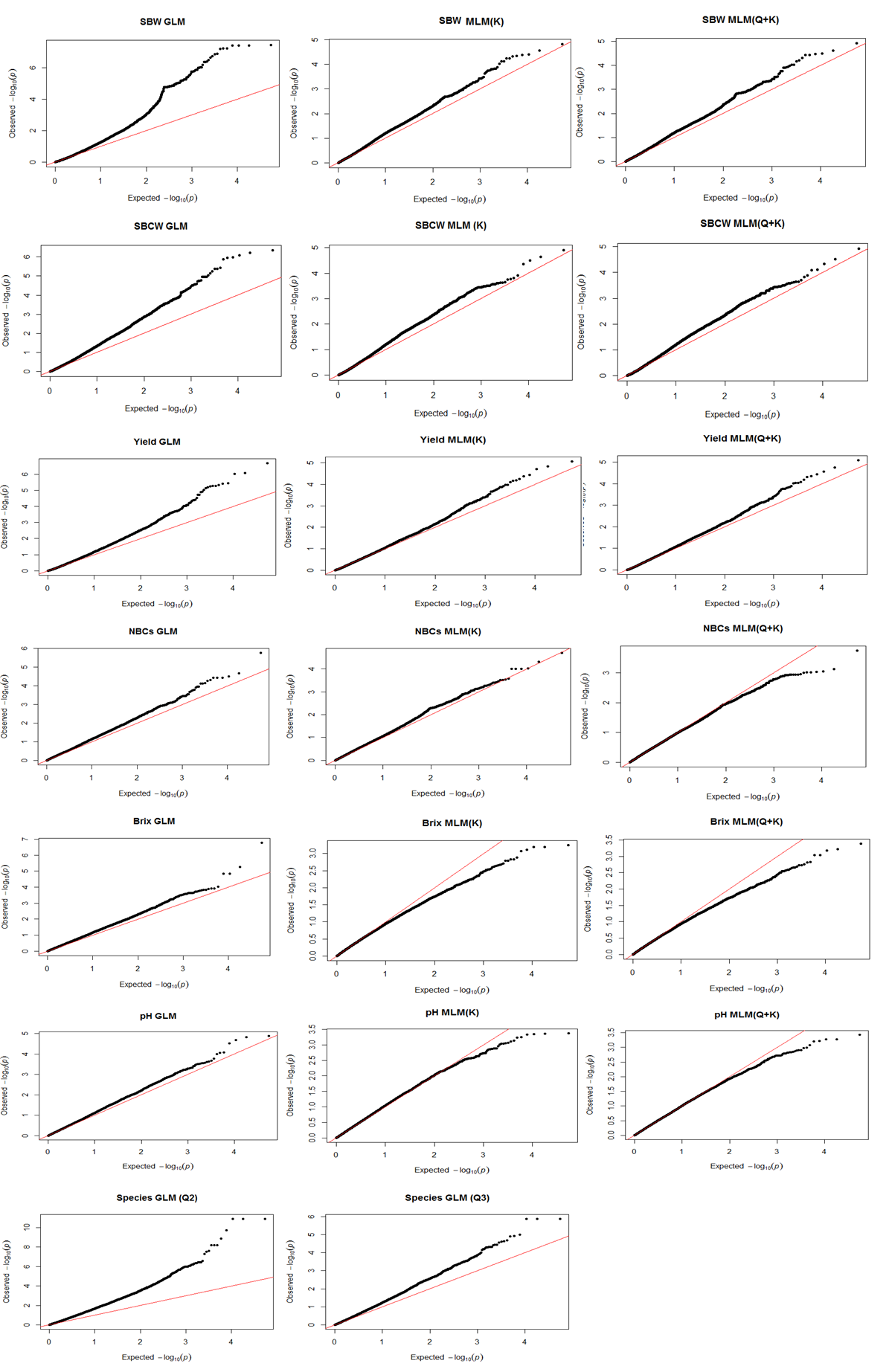


**Figure S9** **:** Manhattan plot of GWAS test for SBW, SBCW, and NBCs. Chr20 includes those SNPs from unassembled regions of the grapevine reference genome.


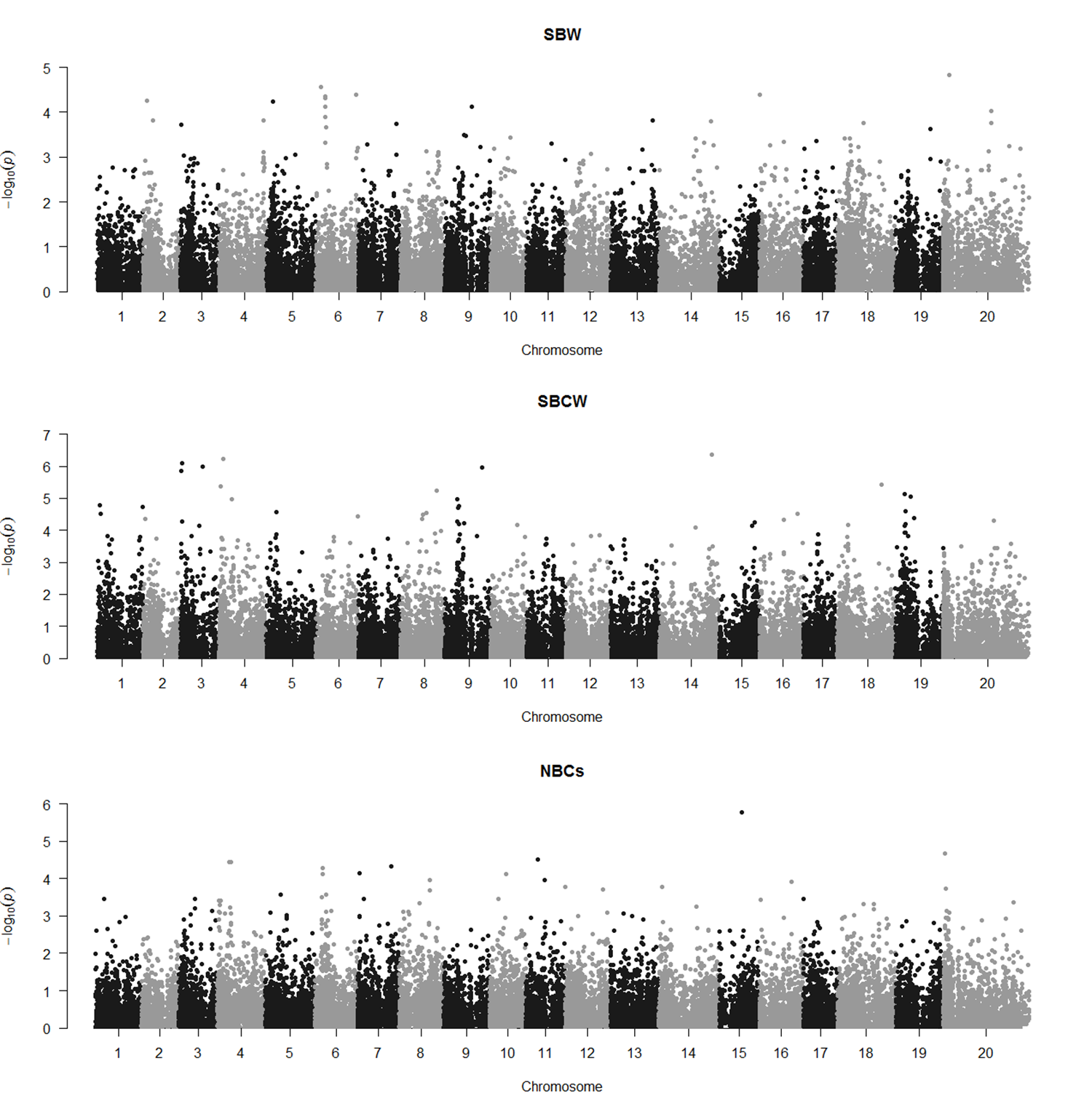


**Figure S10** **:** Manhattan plot of GWAS test for yield, Brixº, and pH (2012). Chr20 includes those SNPs from unassembled regions of the grapevine reference genome.


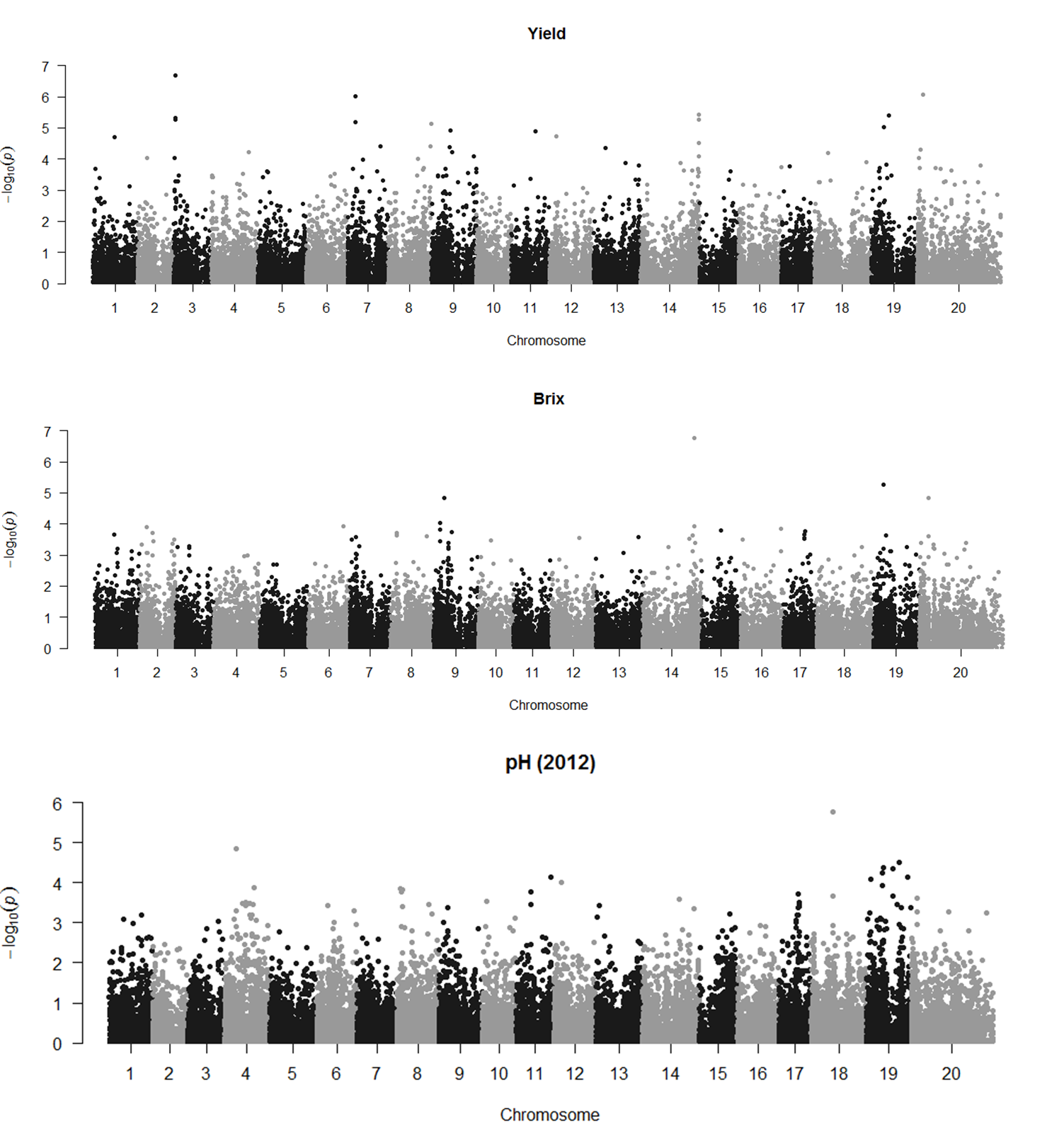


**Figure S11** **:** Manhattan plot of GWAS test for “Species” trait. Chr20 includes those SNPs from unassembled regions of the grapevine reference genome.


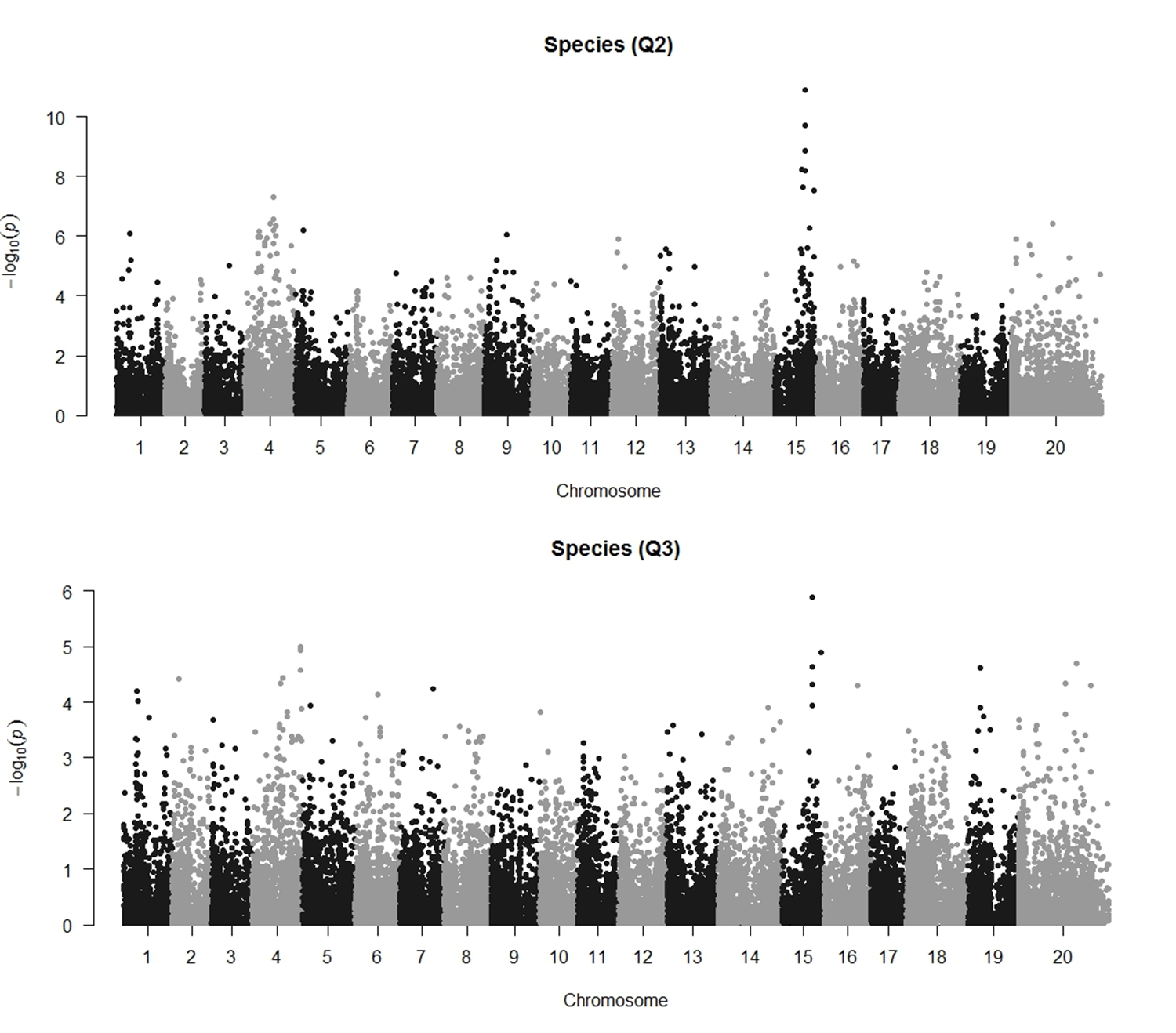


**Figure S12** **:** differences in berry size (2012, 2013, BLUP 2 years) between the three genotypes AA (0), AB (1) and BB (2) of the two most associated SNPs with SBW (g) on chr6.


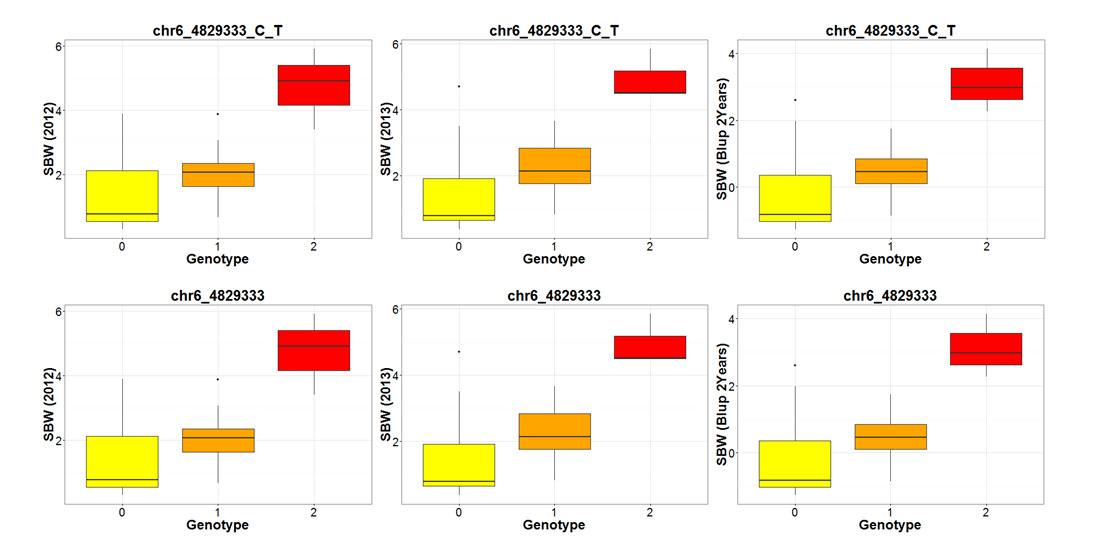


**Figure S13** **:** differences in Brixº (BLUP 2 years) between the three genotypes AA (0), AB (1) and BB (2) of the SNP most associated to Brixº on chr14.


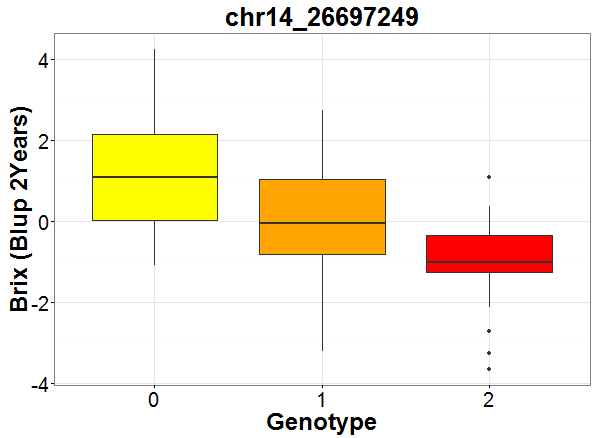


**Table S1:** List of the wild and cultivated accessions used in the present study. “True-to-type” varieties have the accession name marked in bold. The country of origin is indicated only for the *sylvestris* and “true-to-type” cultivars.

| **Sample ID** | **Specie** | **Accession name** | **Accession Number** | **Country of origin** | **Samples Removed for a missing rate > 0.2** |
| --- | --- | --- | --- | --- | --- |
| sample_01 | sativa | Alba aganin isyoum | 61 |  |  |
| sample_02 | sativa | **Alarije** | 40 | Spain |  |
| sample_03 | sativa | **Arnsburger** | 597 | Germany |  |
| sample_04 | sativa | **Brustiano** | 2636 | Italy |  |
| sample_05 | sativa | **Forsellina** | 747 | Italy |  |
| sample_06 | sativa | **Gewuerztraminer** | 1701 | Italy |  |
| sample_07 | sativa | **Leon Millot** | 704 | France |  |
| sample_08 | sativa | Beli Medenac | 198 |  |  |
| sample_09 | sativa | **Macabeu** | 1119 | Spain |  |
| sample_10 | sativa | **Mornen noir** | 1287 | France |  |
| sample_11 | sativa | **Lambrusco casetta** | 2457 | Italy |  |
| sample_12 | sativa | **Corbera** | 2518 | Italy |  |
| sample_13 | sativa | **Reze** | 1673 | Switzerland | **√** |
| sample_14 | sativa | **Roussanne** | 1716 | France |  |
| sample_15 | sativa | **Csaba gyongye** | 608 | Hungary |  |
| sample_16 | sativa | **Saperavi** | 1757 | Georgia |  |
| sample_17 | sativa | **Malvasia Istriana** | 1180 | Italy |  |
| sample_18 | sativa | **Jacquere** | 996 | France |  |
| sample_19 | sativa | **Zilavka** | 2430 | Bosnia-Herzegovina |  |
| sample_20 | sativa | **Vernaccia di S.Gimignano** | 2324 | Italy |  |
| sample_21 | sativa | **Shiraz** | 1829 | France |  |
| sample_22 | sativa | **Claverie coulard** | 475 | France |  |
| sample_23 | sativa | **Ak chekerek** | 58 | Turkmenistan |  |
| sample_24 | sativa | **Ortrugo** | 1414 | Italy |  |
| sample_25 | sativa | **Limnio** | 1112 | Greece |  |
| sample_26 | sativa | **Canorroio** | 332 | Spain |  |
| sample_27 | sativa | **Pinot Noir** | 1577 | France |  |
| sample_28 | sativa | **Verdelet** | 2667 | France |  |
| sample_29 | sativa | **Pignoletto** | 910 | Italy |  |
| sample_30 | sativa | Aris | 121 |  |  |
| sample_31 | sativa | Nevado | 1390 |  | **√** |
| sample_32 | sativa | Moscato | 2631 |  |  |
| sample_33 | sativa | Piè di Palombo | 2634 |  |  |
| sample_34 | sativa | Rossola | 1706 |  |  |
| sample_35 | sativa | Castor | 352 |  |  |
| sample_36 | sativa | Armenia chi 10 | 128 |  |  |
| sample_37 | sativa | Trollinger Rot | 1946 |  |  |
| sample_38 | sativa | Espadeiro blanco | 707 |  |  |
| sample_39 | sativa | Muscat Bleu | 2664 |  |  |
| sample_40 | sativa | Bracciola nera | 262 |  |  |
| sample_41 | sativa | Semidano | 1816 |  |  |
| sample_42 | sativa | Soleil Blanc | 2671 |  | **√** |
| sample_43 | sativa | Buffalo | 2672 |  |  |
| sample_44 | sativa | Ak ouzioum tagapskii | 59 |  |  |
| sample_45 | sativa | Ahmed | 50 |  |  |
| sample_46 | sativa | V.berlandieri Colombard | 1975 |  |  |
| sample_47 | sativa | V,silvestris Lauri 2 | 2147 |  | **√** |
| sample_48 | sativa | V,silvestris cl, Guemuld 103-64 | 2107 |  |  |
| sample_49 | sylvestris |  | 2152 | Italy |  |
| sample_50 | sylvestris |  | 2181 | Italy |  |
| sample_51 | sylvestris |  | 2257 | Italy |  |
| sample_52 | sylvestris |  | 2183 | Italy |  |
| sample_53 | sylvestris |  | 2182 | Italy |  |
| sample_54 | sylvestris |  | 2187 | Italy |  |
| sample_55 | sylvestris |  | 2185 | Italy |  |
| sample_56 | sylvestris |  | 2163 | Italy |  |
| sample_57 | sylvestris |  | 2165 | Italy |  |
| sample_58 | sylvestris |  | 2162 | Italy | **√** |
| sample_59 | sylvestris |  | 2145 | France |  |
| sample_60 | sylvestris |  | 2219 | Italy |  |
| sample_61 | sylvestris |  | 2144 | France |  |
| sample_62 | sylvestris |  | 2129 | Italy |  |
| sample_63 | sylvestris |  | 2071 | Italy |  |
| sample_64 | sylvestris |  | 2169 | Italy |  |
| sample_65 | sylvestris |  | 2264 | Italy |  |
| sample_66 | sylvestris |  | 2261 | Italy |  |
| sample_67 | sylvestris |  | 2252 | Italy |  |
| sample_68 | sylvestris |  | 2204 | Italy |  |
| sample_69 | sylvestris |  | 2194 | Italy |  |
| sample_70 | sylvestris |  | 2214 | Italy |  |
| sample_71 | sylvestris |  | 2119 | Germany | **√** |
| sample_72 | sylvestris |  | 2122 | Germany |  |
| sample_73 | sylvestris |  | 2178 | Italy |  |
| sample_74 | sylvestris |  | 2275 | Italy |  |
| sample_75 | sylvestris |  | 2134 | Italy |  |
| sample_76 | sylvestris |  | 2117 | Germany |  |
| sample_77 | sylvestris |  | 2244 | Italy |  |
| sample_78 | sylvestris |  | 2143 | Germany |  |
| sample_79 | sylvestris |  | 2236 | Italy |  |
| sample_80 | sylvestris |  | 2127 | Italy |  |
| sample_81 | sylvestris |  | 2138 | Italy |  |
| sample_82 | sylvestris |  | 2198 | Italy |  |
| sample_83 | sylvestris |  | 2150 | Italy |  |
| sample_84 | sylvestris |  | 2200 | Italy |  |
| sample_85 | sylvestris |  | 2268 | Italy |  |
| sample_86 | sylvestris |  | 2216 | Italy |  |
| sample_87 | sylvestris |  | 2190 | Italy |  |
| sample_88 | sylvestris |  | 2231 | Italy |  |
| sample_89 | sylvestris |  | 2233 | Italy |  |
| sample_90 | sylvestris |  | 2171 | Italy |  |
| sample_91 | sylvestris |  | 2172 | Italy |  |
| sample_92 | sylvestris |  | 2132 | Italy |  |

**Table S2:** Ancestry values inferred by fastSTRUCTURE for 44 grapevine cultivars and 42 wild individuals genotyped at 26,893 SNP loci. The three subgroups inferred based on a membership cutoff of 0.75 are highlighted in grey.

| **Sample ID** | **Accession Name** | **Population** | **Cluster membership** | | |
| --- | --- | --- | --- | --- | --- |
|  |  |  | K1 | K2 | K3 |
| sample_52 |  | sylvestris | 1 | 0 | 0 |
| sample_53 |  | sylvestris | 1 | 0 | 0 |
| sample_54 |  | sylvestris | 1 | 0 | 0 |
| sample_55 |  | sylvestris | 1 | 0 | 0 |
| sample_59 |  | sylvestris | 1 | 0 | 0 |
| sample_60 |  | sylvestris | 1 | 0 | 0 |
| sample_61 |  | sylvestris | 1 | 0 | 0 |
| sample_62 |  | sylvestris | 1 | 0 | 0 |
| sample_65 |  | sylvestris | 1 | 0 | 0 |
| sample_72 |  | sylvestris | 1 | 0 | 0 |
| sample_74 |  | sylvestris | 1 | 0 | 0 |
| sample_81 |  | sylvestris | 1 | 0 | 0 |
| sample_78 |  | sylvestris | 0.97 | 0.03 | 0 |
| sample_80 |  | sylvestris | 0.95 | 0 | 0.05 |
| sample_67 |  | sylvestris | 0.94 | 0 | 0.06 |
| sample_75 |  | sylvestris | 0.94 | 0 | 0.06 |
| sample_51 |  | sylvestris | 0.93 | 0 | 0.07 |
| sample_76 |  | sylvestris | 0.93 | 0.07 | 0 |
| sample_57 |  | sylvestris | 0.91 | 0 | 0.09 |
| sample_66 |  | sylvestris | 0.91 | 0 | 0.09 |
| sample_73 |  | sylvestris | 0.91 | 0 | 0.09 |
| sample_79 |  | sylvestris | 0.91 | 0 | 0.09 |
| sample_88 |  | sylvestris | 0.91 | 0 | 0.09 |
| sample_77 |  | sylvestris | 0.9 | 0 | 0.1 |
| sample_70 |  | sylvestris | 0.89 | 0 | 0.11 |
| sample_63 |  | sylvestris | 0.88 | 0 | 0.12 |
| sample_90 |  | sylvestris | 0.86 | 0 | 0.14 |
| sample_50 |  | sylvestris | 0.82 | 0 | 0.18 |
| sample_64 |  | sylvestris | 0.81 | 0 | 0.19 |
| sample_84 |  | sylvestris | 0.8 | 0 | 0.2 |
| sample_86 |  | sylvestris | 0.8 | 0 | 0.2 |
| sample_82 |  | sylvestris | 0.79 | 0 | 0.21 |
| sample_69 |  | sylvestris | 0.78 | 0 | 0.22 |
| sample_89 |  | sylvestris | 0.78 | 0 | 0.22 |
| sample_91 |  | sylvestris | 0.78 | 0 | 0.22 |
| sample_49 |  | sylvestris | 0.77 | 0 | 0.23 |
| sample_27 | Pinot Noir | sativa | 0 | 1 | 0 |
| sample_06 | Gewuerztraminer | sativa | 0.08 | 0.92 | 0 |
| sample_10 | Mornan Noir | sativa | 0.08 | 0.89 | 0.03 |
| sample_01 | Alba aganyn isyoum | sativa | 0 | 0 | 1 |
| sample_02 | Alarije | sativa | 0 | 0 | 1 |
| sample_19 | Zilavka | sativa | 0 | 0 | 1 |
| sample_23 | Ak chekerek | sativa | 0 | 0 | 1 |
| sample_25 | Limnio | sativa | 0 | 0 | 1 |
| sample_26 | Canorroio | sativa | 0 | 0 | 1 |
| sample_34 | Rossola | sativa | 0 | 0 | 1 |
| sample_36 | Armenia chi 10 | sativa | 0 | 0 | 1 |
| sample_37 | Trollinger Rot | sativa | 0 | 0 | 1 |
| sample_44 | Ak ouzioum tagapskii | sativa | 0 | 0 | 1 |
| sample_45 | Ahmed | sativa | 0 | 0 | 1 |
| sample_04 | Brustiano | sativa | 0.02 | 0 | 0.98 |
| sample_20 | Vernaccia di S.Gimignano | sativa | 0.04 | 0 | 0.96 |
| sample_08 | Beli Medenac | sativa | 0.06 | 0 | 0.94 |
| sample_09 | Macabeu | sativa | 0.06 | 0 | 0.94 |
| sample_16 | Saperavi | sativa | 0.06 | 0 | 0.94 |
| sample_17 | Malvasia Istriana | sativa | 0.08 | 0 | 0.92 |
| sample_33 | Piè di Palombo | sativa | 0.09 | 0 | 0.91 |
| sample_43 | Buffalo | sativa | 0.1 | 0 | 0.9 |
| sample_39 | Muscat Bleu | sativa | 0 | 0.12 | 0.88 |
| sample_32 | Moscato | sativa | 0.13 | 0 | 0.87 |
| sample_38 | Espadeiro blanco | sativa | 0.13 | 0 | 0.87 |
| sample_29 | Pignoletto | sativa | 0.15 | 0 | 0.85 |
| sample_46 | V.berlandieri Colombard | sativa | 0.15 | 0 | 0.85 |
| sample_28 | Verdelet | sativa | 0.17 | 0 | 0.83 |
| sample_18 | Jacquere | sativa | 0.18 | 0 | 0.82 |
| sample_35 | Castor | sativa | 0.12 | 0.07 | 0.81 |
| sample_30 | Aris | sativa | 0.21 | 0 | 0.79 |
| sample_40 | Bracciola nera | sativa | 0.05 | 0.21 | 0.73 |
| sample_14 | Roussanne | sativa | 0.27 | 0 | 0.73 |
| sample_24 | Ortrugo | sativa | 0.18 | 0.1 | 0.71 |
| sample_07 | Leon Millot | sativa | 0.17 | 0.19 | 0.64 |
| sample_12 | Corbera | sativa | 0.09 | 0.28 | 0.63 |
| sample_48 | V,silvestris cl, Guemuld 103-64 | sativa | 0.39 | 0 | 0.61 |
| sample_05 | Forsellina | sativa | 0.29 | 0.16 | 0.55 |
| sample_15 | Csaba gyongye | sativa | 0 | 0.47 | 0.53 |
| sample_41 | Semidano | sativa | 0.03 | 0.45 | 0.52 |
| sample_83 |  | sylvestris | 0.48 | 0 | 0.52 |
| sample_21 | Shiraz | sativa | 0.29 | 0.35 | 0.36 |
| sample_11 | Lambrusco casetta | sativa | 0.48 | 0.18 | 0.35 |
| sample_56 |  | sylvestris | 0.67 | 0 | 0.33 |
| sample_87 |  | sylvestris | 0.67 | 0 | 0.33 |
| sample_92 |  | sylvestris | 0.68 | 0 | 0.32 |
| sample_03 | Arnsburger | sativa | 0 | 0.71 | 0.29 |
| sample_68 |  | sylvestris | 0.73 | 0 | 0.27 |
| sample_85 |  | sylvestris | 0.75 | 0 | 0.25 |
| sample_22 | Claverie coulard | sativa | 0.45 | 0.39 | 0.16 |

**Table S3:** Grapevine genes included in the enriched functional classes significantly differentiated between *sativa* and *sylvestris* accessions (significance cutoffs: **99^th^ percentile; *95^th^ percentile).

| **Gene ID** | **Chr** | **Position** | **Fst** | **GO Term** | **Gene name** | **Gene Annotation (v2.1)** |
| --- | --- | --- | --- | --- | --- | --- |
| VIT_204s0008g00050 | **4** | 16202:16771 | **0.49**** | nitrogen compound metabolic process | RPL5B | ribosomal protein |
| VIT_204s0008g03840 | **4** | 3182895:3185997 | **0.45**** | response to biotic stimulus | - | ankyrin repeat-containing protein |
| VIT_204s0008g03830 | **4** | 3178460:3182280 | **0.45**** | organic substance metabolic process | RBL11 | rhomboid family protein |
| VIT_208s0040g01150 | **8** | 12159018:12162600 | **0.42**** | carbohydrate metabolic process | CPN10 | 10 kda chaperonin |
| VIT_217s0000g05240 | **17** | 5737662:5753467 | **0.4**** | response to abscisic acid | - | nuclear transport factor 2 and rna recognition motif domain-containing protein |
| VIT_211s0065g00150 | **11** | 13509591:13529415 | **0.4**** | carbohydrate metabolic process | SS4 | soluble starch synthase iv-1 |
| VIT_204s0008g00480 | **4** | 415618:417970 | **0.39**** | nitrogen compound metabolic process | LPA66 | pentatricopeptide repeat-containing protein chloroplastic-like |
| VIT_205s0049g00250 | **5** | 7334595:7335474 | **0.39**** | oxidation-reduction process | - | Desacetoxyvindoline 4-hydroxylase |
| VIT_204s0079g00530 | 4 | 11130821:11137310 | 0.38** | carbohydrate metabolic process | cICDH | nadp-isocitrate dehydrogenase |
| VIT_208s0040g00270 | 8 | 11213199:11217147 | 0.37** | nitrogen compound metabolic process | - | splicing factor 3b subunit 1-like |
| VIT_204s0008g01360 | **4** | 1114709:1118921 | **0.37**** | organic substance metabolic process | - | u-box domain-containing protein 35-like |
| VIT_217s0000g01100 | **17** | 769342:770298 | **0.37**** | organic substance metabolic process | FATB | myristoyl-acyl carrier protein chloroplastic-like |
| VIT_218s0001g05250 | **18** | 4220268:4222313 | **0.37**** | response to salt stress | RAP2 | ap2 erf domain-containing transcription factor |
| VIT_215s0021g01590 | 15 | 12223557:12224200 | 0.37** | defense response | ERF2 | erf2 transcription factor |
| VIT_206s0004g06420 | 6 | 7163946:7166617 | 0.35* | nitrogen compound metabolic process | - | probable lrr receptor-like serine threonine-protein kinase at1g56140-like |
| VIT_206s0004g04180 | 6 | 5152464:5153142 | 0.35* | response to salt stress | - | nucleic acid binding |
| VIT_206s0004g06890 | 6 | 7615724:7620455 | 0.35* | stomatal movement | KT1 | potassium transporter 1-like |
| VIT_217s0000g05270 | 17 | 5761030:5764757 | 0.34* | organic substance metabolic process | - | uncharacterized protein |
| VIT_206s0004g07820 | 6 | 8601569:8603477 | 0.33* | nitrogen compound metabolic process | OTP82 | pentatricopeptide repeat-containing protein at1g08070-like |
| VIT_201s0127g00190 | 1 | 7536963:7537993 | 0.33* | organic substance metabolic process | CRK2 | cysteine-rich receptor-like protein kinase 2 |
| VIT_214s0171g00140 | 14 | 26008741:26011003 | 0.33* | organic substance metabolic process | - | type receptor kinase |
| VIT_206s0004g06980 | 6 | 7687431:7690514 | 0.33* | organic substance metabolic process | - | probable phytol kinase chloroplastic-like |
| VIT_211s0103g00110 | 11 | 15613800:15614090 | 0.33* | oxidation-reduction process | - | photosystem II protein D2 |
| VIT_217s0000g01040 | 17 | 744117:746348 | 0.33* | response to abscisic acid | HSD7 | protein |
| VIT_213s0101g00050 | 13 | 11498528:11499084 | 0.32* | nitrogen compound metabolic process | RPS1 | ribosomal protein s1 |
| VIT_217s0000g06390 | 17 | 6970923:6972402 | 0.32* | nitrogen compound metabolic process | - | uncharacterized protein |
| VIT_208s0056g01650 | 8 | 2648151:2649186 | 0.32* | response to biotic stimulus | LBD20 | protein |
| VIT_208s0105g00480 | 8 | 7811452:7821404 | 0.32* | carbohydrate metabolic process | SAC8 | transmembrane protein g5p |
| VIT_210s0042g00290 | 10 | 13123742:13128460 | 0.32* | organic substance metabolic process | SMO1-3 | protein |
| VIT_212s0059g01590 | 12 | 6491575:6511602 | 0.32* | organic substance metabolic process | - | gdsl esterase lipase |
| VIT_205s0049g00410 | 5 | 7455371:7457433 | 0.32* | oxidation-reduction process | - | 1-aminocyclopropane-1-carboxylate oxidase homolog 1 |
| VIT_208s0007g05410 | 8 | 19354822:19363364 | 0.31* | nitrogen compound metabolic process | CBL | cystathionine beta-lyase |
| VIT_207s0141g00580 | 7 | 324514:331958 | 0.31* | carbohydrate metabolic process | GAUT6 | alpha- -galacturonosyltransferase |
| VIT_214s0066g00600 | 14 | 27087956:27088713 | 0.31* | organic substance metabolic process | - | uncharacterized protein |
| VIT_217s0000g00170 | 17 | 88282:89690 | 0.31* | organic substance metabolic process | VIM1 | zinc finger |
| VIT_207s0129g00680 | 7 | 15918217:15921201 | 0.31* | organic substance metabolic process | - | pentatricopeptide repeat-containing protein chloroplastic-like |
| VIT_213s0156g00150 | 13 | 23889012:23889722 | 0.31* | oxidation-reduction process | - | protein |
| VIT_211s0016g02340 | 11 | 1886162:1888997 | 0.3* | nitrogen compound metabolic process | CDA1 | cytidine deaminase |
| VIT_217s0000g04710 | 17 | 5104509:5107597 | 0.3* | nitrogen compound metabolic process | - | pentatricopeptide repeat-containing protein |
| VIT_206s0004g05500 | 6 | 6342667:6344806 | 0.3* | nitrogen compound metabolic process | - | myosin heavy chain-related protein |
| VIT_205s0102g00773 | 5 | 22709504:22709776 | 0.3* | defense response | - | probable disease resistance protein rdl6 rf9-like |
| VIT_208s0040g03200 | 8 | 14139778:14141968 | 0.29* | methylation | - | 60s ribosomal protein l4-1 |
| VIT_205s0020g03060 | 5 | 4794345:4798018 | 0.29* | nitrogen compound metabolic process | CYCT1-4 | cyclin t1 |
| VIT_205s0020g03070 | 5 | 4800109:4802663 | 0.29* | nitrogen compound metabolic process | - | cyclin family protein |
| VIT_208s0040g03290 | 8 | 14236327:14250749 | 0.29* | nitrogen compound metabolic process | MCM8 | dna replication licensing factor mcm8-like |
| VIT_206s0009g03385 | 6 | 16617200:16618027 | 0.29* | response to biotic stimulus | - | protein |
| VIT_201s0026g00090 | 1 | 8711856:8728580 | 0.29* | carbohydrate metabolic process | ULP1D | ubiquitin-like-specific protease 1c |
| VIT_201s0026g00100 | 1 | 8738934:8750115 | 0.29* | carbohydrate metabolic process | ULP1D | ulp1 protease |
| VIT_206s0004g08080 | 6 | 8843648:8845413 | 0.29* | carbohydrate metabolic process | XLG1 | - |
| VIT_208s0007g03030 | 8 | 17078610:17080600 | 0.29* | methylation | UBQ1 | ubiquitin fusion protein |
| VIT_211s0065g00640 | 11 | 14530232:14586262 | 0.29* | organic substance metabolic process | CAS1 | cycloartenol synthase |
| VIT_217s0000g00070 | 17 | 35355:36590 | 0.29* | organic substance metabolic process | - | protein |
| VIT_219s0085g00190 | 19 | 22516711:22517184 | 0.29* | organic substance metabolic process | SK4 | skp1-like protein |
| VIT_219s0085g00195 | 19 | 22520035:22520493 | 0.29* | organic substance metabolic process | SK4 | skp1-like protein |
| VIT_205s0029g00180 | 5 | 14365888:14385337 | 0.29* | organic substance metabolic process | ERD2B | ER lumen protein retaining receptor-like |
| VIT_206s0004g05610 | 6 | 6420422:6423513 | 0.29* | organic substance metabolic process | - | subtilisin-like serine endopeptidase family protein |
| VIT_208s0007g08780 | 8 | 22158694:22159606 | 0.29* | organic substance metabolic process | MIZ1 | uncharacterized protein |
| VIT_214s0066g02170 | 14 | 28394896:28398366 | 0.29* | oxidation-reduction process | - | prolyl 4-hydroxylase |
| VIT_208s0040g03180 | 8 | 14133347:14136582 | 0.29* | oxidation-reduction process | RAP2 | ap2 domain-containing transcription factor |
| VIT_214s0066g02040 | 14 | 28307901:28309789 | 0.29* | response to abscisic acid | AATP1 | atp binding |
| VIT_214s0066g02050 | 14 | 28314798:28316327 | 0.29* | response to abscisic acid | - | protein |
| VIT_214s0066g02060 | 14 | 28318004:28319797 | 0.29* | response to abscisic acid | AATP1 | atp binding |
| VIT_214s0066g02100 | 14 | 28353929:28355637 | 0.29* | response to abscisic acid | AATP1 | mitochondrial chaperone bcs1 |
| VIT_214s0066g02110 | 14 | 28358493:28360078 | 0.29* | response to abscisic acid | AATP1 | atp binding |
| VIT_213s0067g03350 | 13 | 1837463:1838505 | 0.28* | methylation | - | 60s ribosomal protein l4-1 |
| VIT_206s0004g03730 | 6 | 4680671:4687232 | 0.28* | nitrogen compound metabolic process | NRPC1 | dna-directed rna polymerase iii subunit rpc1-like |
| VIT_206s0004g03740 | 6 | 4692715:4737715 | 0.28* | nitrogen compound metabolic process | NRPC1 | dna-directed rna polymerase iii subunit rpc1-like |
| VIT_206s0004g03780 | 6 | 4759942:4760340 | 0.28* | nitrogen compound metabolic process | PRS | wuschel-related homeobox 3 |
| VIT_206s0004g04040 | 6 | 5021170:5031333 | 0.28* | nitrogen compound metabolic process | - | pentatricopeptide repeat-containing protein |
| VIT_206s0004g05930 | 6 | 6666460:6669570 | 0.28* | nitrogen compound metabolic process | PCNA2 | proliferating cell nuclear antigen |
| VIT_208s0007g03340 | 8 | 17307190:17311533 | 0.28* | nitrogen compound metabolic process | - | ribosomal protein l1 |
| VIT_215s0046g01190 | 15 | 18217371:18218971 | 0.28* | regulation of plant-type hypersensitive response | GT72B1 | hydroquinone glucosyltransferase |
| VIT_215s0046g01210 | 15 | 18226980:18228562 | 0.28* | regulation of plant-type hypersensitive response | GT72B1 | hydroquinone glucosyltransferase |
| VIT_204s0023g00110 | 4 | 16084017:16085284 | 0.28* | carbohydrate metabolic process | - | alpha- -glucan-protein synthase |
| VIT_206s0004g05040 | 6 | 5967109:5968595 | 0.28* | methylation | - | isoprenylcysteine carboxyl methyltransferase |
| VIT_215s0021g01110 | 15 | 11136019:11137148 | 0.28* | organic substance metabolic process | CYP714A1 | cytochrome p450 |
| VIT_215s0021g01380 | 15 | 11651026:11665356 | 0.28* | organic substance metabolic process | - | kinase like protein |
| VIT_215s0046g01150 | 15 | 18197371:18198267 | 0.28* | organic substance metabolic process | - | anthocyanidin reductase-like |
| VIT_215s0046g01320 | 15 | 18339430:18341953 | 0.28* | organic substance metabolic process | - | protein kinase-like protein |
| VIT_216s0098g01780 | 16 | 21844781:21851539 | 0.28* | organic substance metabolic process | SSI1 | soluble starch synthase I |
| VIT_217s0000g05592 | 17 | 6117344:6117909 | 0.28* | organic substance metabolic process | - | momilactone a synthase |
| VIT_217s0000g05600 | 17 | 6124001:6125145 | 0.28* | organic substance metabolic process | - | short-chain alcohol dehydrogenase |
| VIT_205s0049g01050 | 5 | 8090670:8091361 | 0.28* | organic substance metabolic process | - | protein |
| VIT_205s0094g01270 | 5 | 24541244:24543738 | 0.28* | organic substance metabolic process | BIR1 | probably inactive leucine-rich repeat receptor-like protein kinase at5g48380-like |
| VIT_207s0005g04840 | 7 | 8105826:8106863 | 0.28* | organic substance metabolic process | MAPKKK21 | mitogen-activated protein kinase kinase kinase anp1-like |
| VIT_207s0005g03750 | 7 | 6715566:6716963 | 0.28* | oxidation-reduction process | RIC7 | protein |
| VIT_207s0005g04060 | 7 | 7161837:7164031 | 0.28* | oxidation-reduction process | - | protein |
| VIT_215s0046g00440 | 15 | 17407010:17409649 | 0.28* | defense response | PI4K | phosphoinositide 4-kinase gamma 4 |
| VIT_211s0016g04700 | 11 | 3988735:3992060 | 0.27055* | organic substance metabolic process | KCS11 | beta-ketoacyl-coa synthase family protein |
| VIT_206s0004g06310 | 6 | 7093095:7104132 | 0.27* | methylation | - | 60s acidic ribosomal protein p0 |
| VIT_216s0022g01860 | 16 | 14100069:14219500 | 0.27* | nucleic acid phosphodiester bond hydrolysis | CPSF160 | protein |
| VIT_204s0008g02230 | 4 | 1834035:1834800 | 0.27* | nitrogen compound metabolic process | - | ap2 erf domain-containing transcription factor |
| VIT_204s0008g03960 | 4 | 3298715:3326185 | 0.27* | nitrogen compound metabolic process | - | protein |
| VIT_211s0016g04580 | 11 | 3888012:3891011 | 0.27* | nitrogen compound metabolic process | CRR21 | chlororespiratory reduction partial |
| VIT_211s0016g04630 | 11 | 3959481:3961177 | 0.27* | nitrogen compound metabolic process | GAI | della protein |
| VIT_211s0016g04640 | 11 | 3966363:3968501 | 0.27* | nitrogen compound metabolic process | GONST4 | gdp-mannose transporter |
| VIT_217s0000g01760 | 17 | 1307825:1308955 | 0.27* | nitrogen compound metabolic process | - | duf246 domain-containing protein at1g04910-like |
| VIT_218s0001g06980 | 18 | 5220072:5221115 | 0.27* | nitrogen compound metabolic process | - | pentatricopeptide repeat-containing protein |
| VIT_205s0020g04520 | 5 | 6332992:6339892 | 0.27* | nitrogen compound metabolic process | LFR | leaf and flower related protein |
| VIT_208s0032g00010 | 8 | 2798984:2799422 | 0.27* | nitrogen compound metabolic process | - | maturase |
| VIT_208s0007g03150 | 8 | 17167523:17169413 | 0.27* | nitrogen compound metabolic process | - | pentatricopeptide repeat-containing protein |
| VIT_209s0070g00360 | 9 | 13521416:13522967 | 0.27* | nitrogen compound metabolic process | - | aryl-alcohol dehydrogenase -like |
| VIT_209s0054g01000 | 9 | 21857946:21860542 | 0.27* | nitrogen compound metabolic process | - | uncharacterized protein |
| VIT_211s0206g00140 | 11 | 7470447:7473588 | 0.27* | carbohydrate metabolic process | SVL4 | glycerophosphoryl diester phosphodiesterase family protein |
| VIT_212s0059g01660 | 12 | 6564094:6572507 | 0.27* | organic substance metabolic process | - | - |
| VIT_214s0066g00170 | 14 | 26743582:26745706 | 0.27* | organic substance metabolic process | CYP724A1 | cytochrome p450 724b1 |
| VIT_209s0070g00320 | 9 | 13450437:13452476 | 0.27* | organic substance metabolic process | - | cyclin-dependent kinase f-4-like |
| VIT_207s0031g00100 | 7 | 16332009:16337077 | 0.27* | oxidation-reduction process | - | 2-oxoglutarate-fe -dependent oxygenase domain-containing protein |
| VIT_204s0008g03950 | 4 | 3287257:3289094 | 0.27* | response to abscisic acid | RD22 | dehydration-responsive protein rd22 |

**Table S4:** Genes reported in literature under QTLs for berry weight, flower sex, and berry skin colour and identified in this study as significantly differentiated between wild and cultivated grapevines.

| **Gene ID** | **Chr** | **Position** | **Gene Annotation** | **Trait** | **F_ST_** | **Reference** |
| --- | --- | --- | --- | --- | --- | --- |
| VIT_201s0150g00460 | 1 | 22826079:22829099 | "XTH5"; xyloglucan endotransglycosylase" | Berry weight | 0.28 | 60 |
| VIT_206s0061g01240 | 6 | 19041829:19047502 | "VvHD2C"; histone deacetylase 2C | Berry weight | 0.26 | 61 |
| VIT_217s0000g05110 | 17 | 5600225:5602640 | "CYP78A10"; cytochrome p450 78a3-like | Berry weight | 0.32 | 62 |
| VIT_211s0016g04630 | 11 | 3959481:3961177 | "GAI"; DELLA protein SLR1-like | Berry weight | 0.27 | 43 |
| VIT_218s0001g14000 | 18 | 12002927:12003389 | auxin-induced protein X10A-like | Berry weight | 0.29 | 43 |
| VIT_218s0001g14030 | 18 | 12073128:12076336 | probable cytokinin riboside 5 -monophosphate phosphoribohydrolase logl6-like | Berry weight | 0.29 | 43 |
| VIT_202s0241g00050 | 2 | 4698823:4704204 | uncharacterized protein | Flower sex | 0.29 | 63 |
| VIT_202s0241g00060 | 2 | 4715393:4718698 | uncharacterized protein | Flower sex | 0.29 | 63 |
| VIT_202s0241g00060 | 2 | 4715393:4718698 | uncharacterized protein | Flower sex | 0.29 | 63 |
| VIT_202s0154g00230 | 2 | 5036984:5037952 | pinus taeda anonymous locus 0_16347_01 genomic sequence | Flower sex | 0.36 | 63 |
| VIT_202s0109g00370 | 2 | 13050602:13056119 | RNA recognition motif-containing protein | Berry Skin color | 0.28 | 64 |
| VIT_202s0109g00380 | 2 | 13057949:13076992 | "STRS1"; dead-box atp-dependent rna helicase 5 | Berry Skin color | 0.28 | 64 |
| VIT_202s0033g00450 | 2 | 14308288:14309480 | "MYB113"; transcription factor MYBA3 | Berry Skin color | 0.28 | 4 |
| VIT_202s0033g00460 | 2 | 14313417:14314479 | "MYB113"; transcription factor MYBA4 | Berry Skin color | 0.36 | 4 |
| VIT_207s0005g04890 | 7 | 8141027:8142187 | "GSTU7"; Glutathione S-transferase 25 | Berry Skin color | 0.28 | 64 |
| VIT_208s0040g01040 | 8 | 12066763:12073699 | "scpl46"; serine carboxypeptidase-like 45-like | Berry Skin color | 0.28 | 64 |

**Table S5:**  Descriptive statistics and comparison of the phenotypic data from *sativa* and *sylvestris* accessions.

| **Specie** | **NBCs** | | **Yield** | | **SBW** | | **SBCW** | | **Brix°** | | **pH** | |
| --- | --- | --- | --- | --- | --- | --- | --- | --- | --- | --- | --- | --- |
|  | **Mean** | **SD** | **Mean** | **SD** | **Mean** | **SD** | **Mean** | **SD** | **Mean** | **SD** | **Mean** | **SD** |
| *sativa* | 14.62 | 8.92 | 1.93 | 1.45 | 2.40 | 1.05 | 154.28 | 110.40 | 18.93 | 2.05 | 3.14 | 0.19 |
| *sylvestris* | 7.77 | 5.30 | 0.16 | 0.18 | 0.65 | 0.20 | 15.07 | 11.58 | 19.65 | 1.93 | 2.93 | 0.20 |

**Table S6:**  Pearson’s correlation analysis between traits within the whole population, and the *sativa* and the *sylvestris* accessions separately.

|  | **NBCs** | **Yield** | **SBW** | **SBCW** | **Brix°** | **pH** |
| --- | --- | --- | --- | --- | --- | --- |
| **NBCs** | - | 0.75** | 0.10 | 0.26 | -0.21 | 0.01 |
| **Yield** | 0.75** | - | 0.38* | 0.73** | -0.02 | 0.17 |
| **SBW** | 0.10 | 0.38* | - | 0.59** | -0.31 | 0.01 |
| **SBCW** | 0.26 | 0.73** | 0.59** | - | 0.09 | 0.18 |
| **Brix°** | -0.21 | -0.02 | -0.31 | 0.09 | - | 0.30 |
| **pH** | 0.01 | 0.17 | 0.01 | 0.18 | 0.30 | - |

**Table S7:** SNPs significantly associated to the six traits analysed, with the corresponding Bonferroni-corrected or FDR p-values. MAF: minor allele frequency. R^2^: the proportion of phenotypic variance explained by the marker. SNPs associated to more traits are underlined.

| **Trait** | **Chr** | **SNP** | **Position** | **Alleles** | **MAF** | **p-value** | **R^2^** |
| --- | --- | --- | --- | --- | --- | --- | --- |
| SBW | 6 | chr6_4829333_C_T | 4829333 | G\A | 0.14 | 0.00 | 0.14 |
| SBW | 6 | chr6_4822590 | 4822590 | T\A | 0.15 | 0.00 | 0.14 |
| SBCW | 14 | chr14_26447823 | 26447823 | C\T | 0.25 | 0.01 | 0.16 |
| SBCW | 4 | chr4_2286974 | 2286974 | G\A | 0.38 | 0.02 | 0.16 |
| SBCW | 3 | chr3_724399_C_T | 724399 | G\A | 0.28 | 0.02 | 0.16 |
| SBCW | 3 | chr3_11296490_A_C | 11296490 | A\C | 0.06 | 0.03 | 0.15 |
| SBCW | 9 | chr9_18755332 | 18755332 | T\C | 0.30 | 0.03 | 0.15 |
| SBCW | 3 | chr3_621609_C_T | 621609 | A\G | 0.27 | 0.04 | 0.15 |
| SBCW (2013) | 19 | chr19_9279384 | 9279384 | C\A | 0.28 | 0.00 | 0.17 |
| Yield | 3 | chr3_621609_C_T | 621609 | A\G | 0.27 | 0.01 | 0.19 |
| Yield | 13_RANDOM | chr13_random_2675668 | 2675668 | A\G | 0.21 | 0.02 | 0.17 |
| Yield | 7 | chr7_4151125_C_T | 4151125 | G\A | 0.23 | 0.03 | 0.17 |
| NBCs | 15 | chr15_11573065_C_T | 11573065 | A\G | 0.41 | 0.05 | 0.20 |
| Brix | 14 | chr14_26697249 | 26697249 | C\T | 0.49 | 0.00 | 0.36 |
| pH (2012) | 18 | chr18_11437074 | 11437074 | A\G | 0.32 | 0.05 | 0.25 |
| Species | 1 | chr1_6322315_A_G | 6322315 | A\G | 0.23 | 0.02 | 0.03 |
| Species | 1 | chr1_6366603_A_G | 6366603 | A\G | 0.23 | 0.02 | 0.03 |
| Species | 4 | chr4_6119158_A_G | 6119158 | G\A | 0.09 | 0.03 | 0.03 |
| Species | 4 | chr4_6801276_C_T | 6801276 | A\G | 0.07 | 0.02 | 0.03 |
| Species | 4 | chr4_6962355 | 6962355 | A\G | 0.09 | 0.03 | 0.03 |
| Species | 4 | chr4_7097309_C_T | 7097309 | A\G | 0.09 | 0.03 | 0.03 |
| Species | 4 | chr4_9423465_A_G | 9423465 | G\A | 0.10 | 0.04 | 0.03 |
| Species | 4 | chr4_9539079 | 9539079 | C\A | 0.10 | 0.04 | 0.03 |
| Species | 4 | chr4_10214943 | 10214943 | A\G | 0.11 | 0.03 | 0.03 |
| Species | 4 | chr4_11771908 | 11771908 | A\T | 0.10 | 0.01 | 0.04 |
| Species | 4 | chr4_11779492 | 11779492 | T\C | 0.10 | 0.01 | 0.04 |
| Species | 4 | chr4_13331268 | 13331268 | C\G | 0.10 | 0.01 | 0.03 |
| Species | 4 | chr4_13542485 | 13542485 | A\G | 0.11 | 0.02 | 0.03 |
| Species | 4 | chr4_13633810 | 13633810 | C\T | 0.13 | 0.00 | 0.04 |
| Species | 4 | chr4_14607996 | 14607996 | T\C | 0.13 | 0.03 | 0.03 |
| Species | 4 | chr4_14622644_A_G | 14622644 | G\A | 0.13 | 0.03 | 0.03 |
| Species | 4 | chr4_14637406 | 14637406 | A\G | 0.14 | 0.01 | 0.04 |
| Species | 4 | chr4_14651154_A_G | 14651154 | A\G | 0.14 | 0.01 | 0.04 |
| Species | 5 | chr5_3968213_G_T | 3968213 | C\A | 0.42 | 0.02 | 0.03 |
| Species | 9 | chr9_10609663 | 10609663 | T\C | 0.06 | 0.02 | 0.03 |
| Species | 12 | chr12_2806062_A_G | 2806062 | A\G | 0.13 | 0.03 | 0.03 |
| Species | 15 | chr15_12863124 | 12863124 | T\G | 0.05 | 0.00 | 0.04 |
| Species | 15 | chr15_12988021 | 12988021 | C\T | 0.05 | 0.00 | 0.04 |
| Species | 15 | chr15_13584268 | 13584268 | C\G | 0.06 | 0.00 | 0.04 |
| Species | 15 | chr15_14467891 | 14467891 | G\C | 0.06 | 0.00 | 0.05 |
| Species | 15 | chr15_14532929 | 14532929 | T\C | 0.06 | 0.00 | 0.05 |
| Species | 15 | chr15_14532954 | 14532954 | G\A | 0.06 | 0.00 | 0.05 |
| Species | 15 | chr15_14532983 | 14532983 | T\C | 0.06 | 0.00 | 0.05 |
| Species | 15 | chr15_14547396 | 14547396 | A\T | 0.07 | 0.00 | 0.04 |
| Species | 15 | chr15_14547453 | 14547453 | A\G | 0.08 | 0.00 | 0.04 |
| Species | 15 | chr15_16809941_A_G | 16809941 | A\G | 0.07 | 0.01 | 0.03 |
| Species | 15 | chr15_18786403 | 18786403 | T\C | 0.09 | 0.00 | 0.04 |
| Species | 18_RANDOM | chr18_random_2214072 | 2214072 | T\A | 0.22 | 0.03 | 0.03 |
| Species | UN | chrUn_19893727 | 19893727 | T\C | 0.10 | 0.01 | 0.04 |

**Table S8:** List of candidate genes functionally annotated. Candidate genes for more traits are underlined.

| **Trait** | **Candidate gene** | **Description** | **Chr** | **Start** | **Stop** |
| --- | --- | --- | --- | --- | --- |
| SBW | VIT_206s0004g03870 | cct motif family protein | 6 | 4819033 | 4820561 |
| SBW | VIT_206s0004g03880 | ribonuclease p subunit rpp30 | 6 | 4822479 | 4826371 |
| SBW | VIT_206s0004g03890 | histone -like | 6 | 4826642 | 4827652 |
| SBW | VIT_206s0004g03900 | calcium-transporting atpase endoplasmic reticulum-type-like | 6 | 4827983 | 4833862 |
| SBW | VIT_206s0004g03910 |  | 6 | 4838289 | 4838956 |
| SBCW | VIT_203s0038g00710 |  | 3 | 614468 | 614930 |
| SBCW | VIT_203s0038g00720 | nadh ubiquinone oxidoreductase b22-like subunit | 3 | 615693 | 621041 |
| SBCW | VIT_203s0038g00730 | 30s ribosomal protein mitochondrial | 3 | 623867 | 624348 |
| SBCW | VIT_203s0038g00740 | epimerase family protein slr1223-like | 3 | 624568 | 632364 |
| SBCW | VIT_203s0038g00750 | ubiquitin fusion degradation 1 | 3 | 631573 | 639607 |
| SBCW | VIT_203s0097g00710 | glutamyl-trna reductase | 3 | 11291816 | 11296993 |
| SBCW | VIT_203s0038g00920 |  | 3 | 722303 | 732914 |
| SBCW | VIT_204s0008g02750 | transcription factor bzip | 4 | 2284121 | 2287115 |
| SBCW | VIT_204s0008g02760 | uncharacterized protein | 4 | 2293948 | 2295399 |
| SBCW | VIT_214s0219g00200 | pentatricopeptide repeat-containing protein | 14 | 26446270 | 26448891 |
| SBCW (2013) | VIT_219s0015g01165 | myb-like protein h-like | 19 | 9268237 | 9268638 |
| SBCW (2013) | VIT_219s0015g01170 | myb-like protein h-like | 19 | 9307511 | 9308113 |
| SBCW (2013) | VIT_219s0015g01180 |  | 19 | 9346610 | 9346837 |
| SBCW (2013) | VIT_219s0015g01190 | ubiquitin-conjugating enzyme e2-17 kda | 19 | 9349102 | 9351951 |
| SBCW (2013) | VIT_219s0015g01200 | Ca^2+^ binding protein | 19 | 9372453 | 9376732 |
| SBCW (2013) | VIT_219s0015g01210 | kh domain-containing protein | 19 | 9375765 | 9384111 |
| Yield | VIT_203s0038g00710 |  | 3 | 614468 | 614930 |
| Yield | VIT_203s0038g00720 | nadh ubiquinone oxidoreductase b22-like subunit | 3 | 615693 | 621041 |
| Yield | VIT_203s0038g00730 | 30s ribosomal protein mitochondrial | 3 | 623867 | 624348 |
| Yield | VIT_203s0038g00740 | epimerase family protein slr1223-like | 3 | 624568 | 632364 |
| Yield | VIT_203s0038g00750 | ubiquitin fusion degradation 1 | 3 | 631573 | 639607 |
| Yield | VIT_207s0005g01660 | pentatricopeptide repeat-containing protein | 7 | 4152252 | 4157457 |
| NBCs | VIT_215s0021g01330 | nucleoside diphosphate kinase | 15 | 11580827 | 11584731 |
| NBCs | VIT_215s0021g01340 | elmo domain-containing protein a-like | 15 | 11582960 | 11597494 |
| Brix | VIT_214s0066g00130 |  | 14 | 26696660 | 26700054 |
| Brix | *VIT_214s0066g00140* | *rna-binding protein cp31* | *14* | *26710173* | *26714980* |
| Brix | *VIT_214s0066g00170* | *cytochrome p450 724b1* | *14* | *26743582* | *26745706* |
| Brix | *VIT_214s0066g00180* | *gtp-binding protein gb2* | *14* | *26750364* | *26755053* |
| Brix | *VIT_214s0066g00200* | *pentatricopeptide repeat-containing protein* | *14* | *26757686* | *26764619* |
| Brix | *VIT_214s0066g00210* | *sgf29 tudor-like domain-containing protein* | *14* | *26767932* | *26777373* |
| Brix | *VIT_214s0066g00220* | *elongation factor chloroplastic-like* | *14* | *26778091* | *26785349* |
| Brix | *VIT_214s0066g00240* | *gdsl esterase lipase at5g14450-like* | *14* | *26785227* | *26792127* |
| Brix | *VIT_214s0066g00250* | alpha*-l-fucosidase 2* | *14* | *26792509* | *26794228* |
| Brix | *VIT_214s0066g00260* | *surfeit locus protein 2* | *14* | *26798154* | *26802046* |
| Brix | *VIT_214s0066g00270* | *methyltransferase pmt9* | *14* | *26801312* | *26812771* |
| Brix | *VIT_214s0066g00320* | *pseudouridylate synthase transporter* | *14* | *26846999* | *26853881* |
| pH (2012) | *VIT_218s0001g13350* | *peptide transporter* | *18* | *11374221* | *11381621* |
| pH (2012) | *VIT_218s0001g13360* | *auxin-induced protein 5ng4-like* | *18* | *11383969* | *11387408* |
| pH (2012) | *VIT_218s0001g13370* |  | *18* | *11389856* | *11406678* |
| pH (2012) | *VIT_218s0001g13380* | *cysteine proteinase rd19a-like* | *18* | *11409660* | *11420788* |
| pH (2012) | *VIT_218s0001g13400* | *cysteine proteinase rd19a-like* | *18* | *11425469* | *11427625* |
| pH (2012) | VIT_218s0001g13410 | V-type proton ATPase subunit a3 | 18 | 11428696 | 11471713 |
| Species | VIT_201s0011g06540 | phagocytic receptor 1b-like | 1 | 6319409 | 6326110 |
| Species | VIT_201s0011g06550 | salt overly sensitive 1 (SOS1) | 1 | 6337655 | 6393053 |
| Species | VIT_204s0008g06790 | protein | 4 | 6796327 | 6801946 |
| Species | VIT_204s0008g06800 | enhancer of rudimentary | 4 | 6805069 | 6813240 |
| Species | VIT_204s0008g07020 | rieske iron-sulfur protein tic55 | 4 | 7106442 | 7110433 |
| Species | VIT_204s0043g00250 | 60s ribosomal export protein nmd3-like | 4 | 13328815 | 13332882 |
| Species | VIT_204s0043g00255 | cysteine-rich repeat secretory protein 3-like | 4 | 13339644 | 13341675 |
| Species | VIT_204s0043g00300 | tpx2 (targeting protein for xklp2) family protein | 4 | 13532759 | 13535228 |
| Species | VIT_204s0043g00310 | protein | 4 | 13548461 | 13552582 |
| Species | VIT_204s0043g00340 | transcription repressor kan1-like | 4 | 13640745 | 13650521 |
| Species | VIT_204s0043g00690 | two-component response regulator arr22 | 4 | 14598640 | 14599614 |
| Species | VIT_204s0043g00700 | pentatricopeptide repeat-containing | 4 | 14621916 | 14683126 |
| Species | *VIT_204s0043g00710* | *hypoxia up-regulated protein 1-like* | *4* | *14692195* | *14705141* |
| Species | VIT_204s0069g00990 | uncharacterized protein | 4 | 9417125 | 9419932 |
| Species | VIT_204s0069g01000 | uncharacterized transporter sll0355-like | 4 | 9420202 | 9426120 |
| Species | *VIT_204s0079g00760* | *gtp binding protein* | *4* | *11644192* | *11658927* |
| Species | *VIT_204s0079g00780* | *unc93-like protein* | *4* | *11734958* | *11735411* |
| Species | *VIT_204s0079g00790* | *acyl:coa ligase acetate-coa synthetase-like protein* | *4* | *11741762* | *11744384* |
| Species | VIT_205s0020g02240 | at4g15540 dl3810w | 5 | 3961111 | 3965301 |
| Species | VIT_205s0020g02250 | sugar transporter erd6-like 16-like | 5 | 3968917 | 3972241 |
| Species | VIT_209s0002g09030 | low quality protein: patellin-3-like | 9 | 10601730 | 10602386 |
| Species | VIT_209s0002g09040 | protein | 9 | 10602387 | 10602749 |
| Species | VIT_209s0002g09050 | mitochondrial glycoprotein family protein | 9 | 10606585 | 10609112 |
| Species | VIT_212s0028g02110 | uncharacterized protein | 12 | 2805306 | 2810219 |
| Species | VIT_215s0021g02070 | uncharacterized protein | 15 | 12863018 | 12863736 |
| Species | VIT_215s0021g02080 | hypothetical protein VITISV_023274 [Vitis vinifera] | 15 | 12867496 | 12871100 |
| Species | VIT_215s0021g02140 | e3 ubiquitin-protein ligase bre1-like 1-like | 15 | 12988863 | 13025618 |
| Species | VIT_215s0046g01950 | udp-glycosyltransferase 91a1-like | 15 | 18779360 | 18786610 |
| Species | VIT_215s0046g01960 | udp-glycosyltransferase 91a1-like | 15 | 18789361 | 18790868 |
| Species | VIT_215s0048g00330 | udp-d-glucuronate 4-epimerase 2 | 15 | 14467820 | 14469235 |
| Species | VIT_215s0048g00340 | udp-d-glucuronate 4-epimerase 2 | 15 | 14472530 | 14475674 |
| Species | VIT_215s0048g00400 | nitrate transporter -like (NRT1) | 15 | 14533054 | 14533533 |
| Species | VIT_215s0048g00410 | peptide transporter ptr2 | 15 | 14533534 | 14534223 |
| Species | VIT_215s0048g00420 | arginase | 15 | 14534672 | 14540177 |
| Species | VIT_215s0048g00430 | nitroreductase-like protein | 15 | 14543168 | 14547287 |
| Species | VIT_215s0048g00440 | uncharacterized protein | 15 | 14547386 | 14550878 |
| Species | VIT_215s0048g00460 | uncharacterized protein | 15 | 14555715 | 14559865 |
| Species | VIT_215s0048g02670 | uridylate kinase | 15 | 16807997 | 16809606 |
| Species | VIT_215s0048g02680 | protein | 15 | 16813911 | 16817150 |
| Species | VIT_215s0048g02690 | fad-binding domain-containing protein | 15 | 16818133 | 16821558 |

**Notes S1** **Genome-wide association for six domestication-related traits in grapevine.**

**Phenotyping of the whole grapevine population for six domestication-related traits**

Phenotypic evaluation of 2 to 5 replicates per genotype was performed in 2012 and 2013 for all traits as follows: clusters of each fruit-bearing plant were harvested six weeks after véraison for the evaluation of single bunch weight (OIV code number 502; SBCW), single berry weight (OIV code number 503; SBW), yield (OIV code number 504) and number of bunches per plant (NBCs). Juice samples (50 ml) from berries were measured with FTIR (Fourier transform infrared) using a FOSS instrument (FOSS NIRSystems, Oatley, Australia) for measuring total soluble solids (Brix°) and pH. Average values for replicates were used to evaluate the correlation between the two-year measurements. Moreover, Pearson correlation value (R) between each pair of variables was estimated in the whole population and the two subspecies separately with the ‘Hmisc’ v 3.17-3 R package(Harrel & Dupont, 2016). One to six aberrant values were discarded according to traits. Different mixed models were fitted with the lme4 package(Bates *et al.*, 2014) to identify the best fit model for each trait. Different models, from the simplest, based only on general mean and random genotypic effect (G), to the complete one, based on overall mean, random genotypic effect, fixed year effect (Y) and random genotype x year effect (GxY) were compared. Model selection was based on the Bayesian information criterion (BIC). No data transformation of phenotypes was performed. Based on the best-fitted model, genotypic best linear unbiased predictors (BLUPs) were extracted.

The NBCs ranged from 1.6 (accession “Ahmed”) to 38.8 (cv “Pinot Meunier”) in the *sativa* group with an average of 14.6 bunches per plant (Supplementary Table S5). Instead, the *sylvestris* had an average of 7.8 NBCs, ranging from 1 to 25 bunches. Yield (kg), single berry weight (g; SBW) and single bunch weight (g; SBCW) showed a considerable variation between cultivars and wild grapevines. Indeed, grapevine varieties produced on average 1.9 kg of grapes per year with a maximum of 6.7 kg (cv “Zilavka”), while *sylvestris* genotypes had a yield 91.7% smaller (Supplementary Table S5). The SBW as well as the SBCW varied, respectively, by a four- and ten- fold factor (Supplementary Table S5) between *sativa* and *sylvestris* genotypes. The cultivars presented SBW from 5.9 g (accession “Ak ouzioum tagapskii”) to 0.5 g (accession “Aris”), and SBCW from 456.3 g (accession “Rossola”) to 9.9 g (accession “Aris”). Instead, the *sylvestris* showed SBW from 1.3 g to 0.3 g and SBCW from 47.8 g to 1.7 g. While the Brix° showed less variability between cultivated and wild grapevines (Supplementary Fig. 5B), significant differences were observed between the two subspecies for the pH, where the *sylvestris* presented on average lower pH. The phenotypic data sets for the two years were strongly correlated for all traits, notably for SBW and SBCW (Supplementary Fig. S5).

The distributions of phenotypic data in the whole population and the two subspecies for each year are shown in the Supplementary Figure S6-S7. Most traits displayed a continuous variation within the subspecies. However, in the whole population NBCs, SBW, SBCW and yield were bimodal since cultivars and wild genotypes displayed divergent values. Most of the models selected to estimate BLUP included both genotypic and year effects, except for SBW and SBCW, for which year effect was not significant.

**Genome-wide associations**

The first model applied was the General Linear Model (GLM), which takes into account the population structure inferred by fastSTRUCTURE. The second model applied was the Mixed Linear model, which extends the GLM by incorporating a kinship matrix (K) to define the degree of genetic covariance between pairs of individuals. A centred identical-by-state K matrix was estimated in TASSEL v5.0 by using the method of Endelman and Jannink(Endelman & Jannink, 2013). As both population structure and kinship were incorporated, this full model was called MLM (Q + K). Meanwhile, K only model, called MLM (K), which omits the population structure Q from the full model, was also used. For all traits, GLM (with Q-matrix for K = 3 from the analysis with fastSTRUCTURE) was chosen as the best-fitted model, except for SBW where MLM (K) significantly reduced false-positives compared to GLM. Also, for the trait “Species” only GLM was applied using Q-matrix for either K = 2 (GLM-Q2) or K = 3 (GLM-Q3). (Supplementary Fig. S6).

**Supplementary Note References**

**Bates D, Maechler M, Bolker B, Walker S**. **2014**. lme4: Linear mixed models using Eigen and S4. R package version 1.1-7.

**Endelman JB, Jannink J-L**. **2013**. Shrinkage Estimation of the Realized Relationship Matrix. *G3&amp;#58; Genes|Genomes|Genetics* **2**: 1405–1413.

**Harrel FEJ, Dupont C**. **2016**. Hmisc: Harrel Miscellaneous. R package version 3.17-3.
